# Supplementary material for: Solar-driven membrane separation for direct lithium extraction from artificial salt-lake brine
Source: Nat Commun. 2024 Jan 3;15:238. doi: 10.1038/s41467-023-44625-w (PMC10764783; doi:10.1038/s41467-023-44625-w)
Supplement: Supplementary file 1 — Supplementary Information [file 41467_2023_44625_MOESM1_ESM.pdf]

Supplementary information

Solar-driven membrane separation for direct lithium extraction from artificial salt-lake brine

Shenxiang Zhang *et al.*

List of Content

Supplmentary Methods

Supplementary Figure 1-6

Supplementary Table 1-2

Supplementary References

Supplementary methods

1. Chemicals and materials

Polyether sulfone substrate with a pore size of 0.22 μm was purchased from Shanghai ECO Polymer Sci.&Tech. Co., Ltd. (Shanghai, China). Aniline (99%), ammonium persulfate (98%), ammonia solution (25-28%) and sodium dodecylbenzene sulfonate (SDBS) were purchased from Sinopharm Chemical Reagent Co., Ltd. (Beijing, China). Perchloric acid (70-72%), Trimesoyl chloride (TMC, 98%), piperazine (PIP) and sodium dodecyl sulfonate (SDS) were purchased from Sigma-Aldrich. The SWCNT powder (OD: 1–2 nm, length: 5–30 μm, purity: > 95%) was commercially available from Nanjing XFNANO Materials Tech Co., Ltd. (Nanjing, China). Dopamine hydrochloride was purchased from Alfa-Aesar. Lithium chloride (LiCl, >99%), magnesium chloride ($MgCl_2$, >99%), calcium chloride ($CaCl_2$, >99%) and sodium chloride (NaCl, >99%) were all obtained from Sinopharm Chemical Reagent Co., Ltd. (Beijing, China). All the chemicals were used without purification as received.

2. Preparation of SWCNTs film

SWCNTs film was prepared according to the previous report[1]. Firstly, 40 mg SWCNTs were dispersed in 400 mL 1 mg mL^{-1} SDBS solution and sonicated with a cell crusher for 6 h at 10 W. Then, the solution was centrifuged for 30 min at 5870 g, and the supernatant was collected to obtain the SWCNT dispersion solution. To improve the hydrophily of SWCNT, the surface was coated by polydopamine (PD) as following: 20 mg dopamine hydrochloride was added into 200 mL SWCNTs dispersion. After stirring the solution for 1 h at 40 °C, 20 mL 0.1 m tris(hydroxymethyl)aminomethane (Tris) buffer solution (pH=8.5) was added and further reacted for 48 h under 40 °C. Then, the solution was centrifuged at 5870 g for 30 min to obtain PD/SWCNTs dispersion and stored in a refrigerator at 4 °C. SWCNTs film was prepared by filtering 3 mL PD/SWCNTs dispersion with a concentration of around 0.02 mg mL^{-1} onto PES microfiltration membrane (pore size:

0.22 μm). Finally, the membrane was dried in an oven at 60 °C for 30 min.

3. Determination of molecular weight cutoff (MWCO), pore size and pore size distribution of PA membrane

The pore size distribution and MWCO of the PA-m were studied by the rejection rate of a series of neutral organic molecules[2], including glycerol (92 Da, Stokes radii: 0.261 nm), glucose (180 Da, Stokes radii: 0.359 nm), sucrose (342 Da, Stokes radii: 0.462 nm) and raffinose (504 Da, Stokes radii: 0.538 nm). The feed concentration of each species solution was 200 mg L^{-1} and the applied pressure was 4 bar. The value of MWCO is defined as the molecular weight of the neutral molecule at which the rejection equals 90%. The pore size distribution curve is illustrated as a probability density function (PDF) developed based on the following assumptions: (1) There are no steric or hydrodynamic interactions between the organic solutes and the membrane pores. (2) The mean pore size of the polyamide membrane is equal to the Stokes radius of the organic solute with a measured rejection of 50%. (3) The distribution of the membrane pore size is characterized by the geometric standard deviation of the PDF curve, which is the ratio between the Stokes radius with a rejection of 84.13% to that with a rejection of 50%. Based on the rejection of these molecules, the pore size distribution function can be obtained by the following equation (1):

$$\frac{dR(r_p)}{dr_p} = \frac{1}{r_p \ln \sigma_p \sqrt{2\pi}} exp\left[-\frac{(\ln r_p - \ln \mu_p)^2}{2(\ln \sigma_p)^2}\right] \qquad (1)$$

where μ is the mean pore size, σ is the geometric standard deviation of the PDF curve and r is the Stokes radius of the organic solute.

Supplementary data and figure

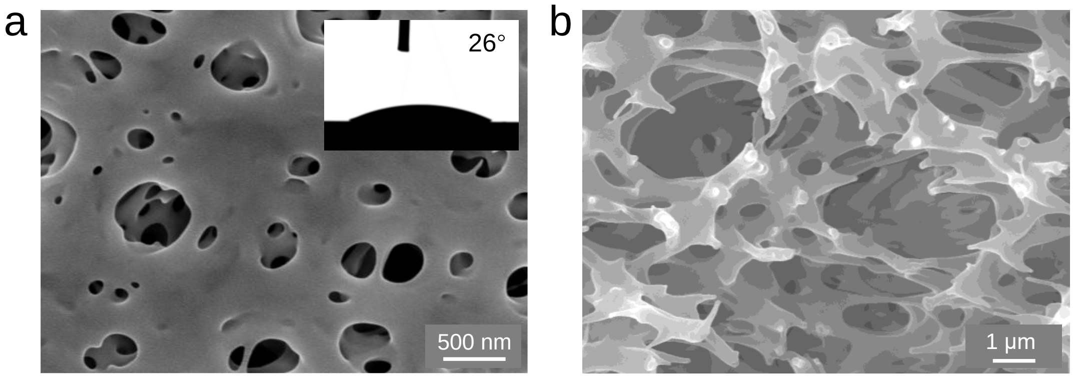

Supplementary Fig. 1 (a) Surface and (b) Cross-sectional SEM image of PES membrane. Inset in Figure (a) shows the instantaneous contact angle of a water drop on PES membrane surface.

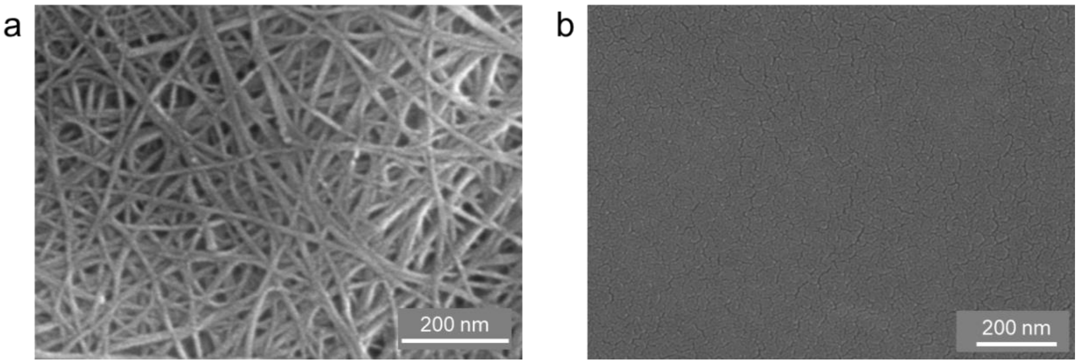

Supplementary Fig. 2 Surface and SEM image of (a) SWCNTs film and (b) PA-2 membrane.

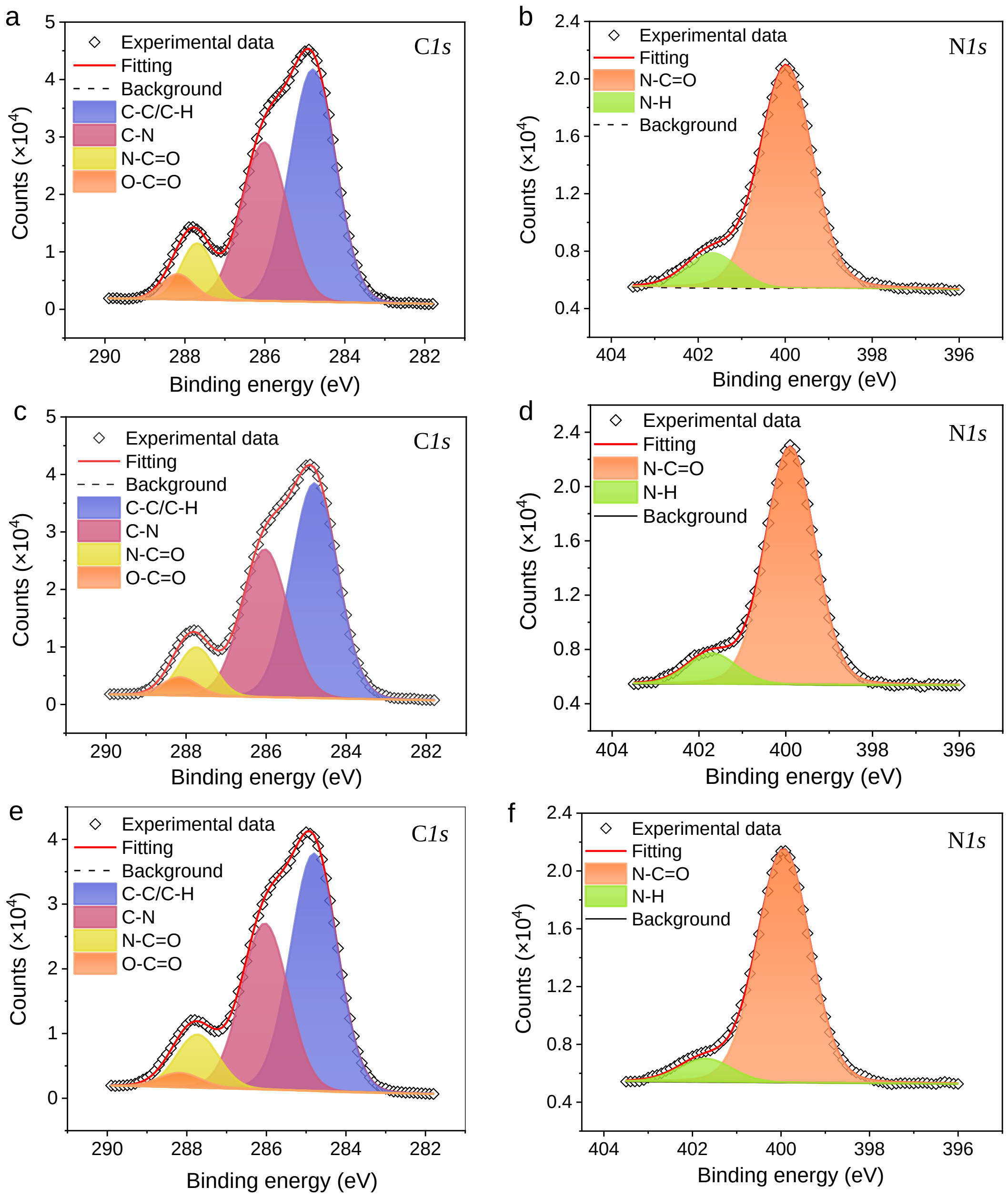

Supplementary Fig. 3 High-resolution XPS spectra of (a and b) PA-1, (c and d) PA-2, (e and f) PA-4 membrane. (a, c and e) C*1s* spectra of PA membranes. (b, d, and f) N*1s* spectra of PA membranes. Three C*1s* peaks were detected, one at 284.8 eV (aliphatic/aromatic C-H or C-C bonds), the second one at 286.0 eV (C-N), the third one at 287.7 eV (amide O=C-N) and the other at 288.5 eV (carboxy O=C-O groups). Two N*1s* signals were observed, peak one at 400.0 eV (N-C=O) and peak two at 401.7 eV (R-N-H).

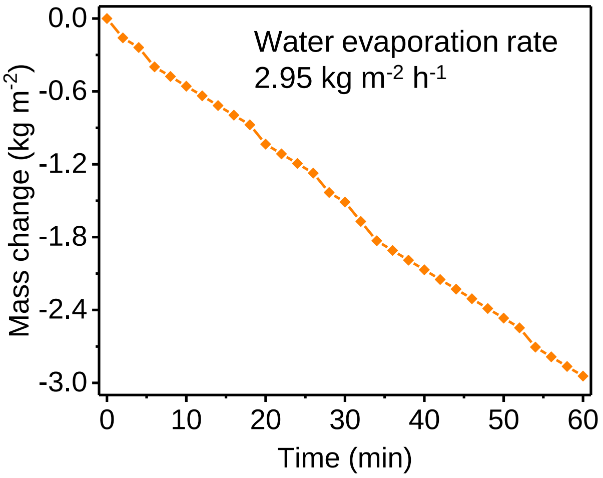

Supplementary Fig. 4 Water evaporation rate when processing a mixed salt solution under 3 sun irradiation.

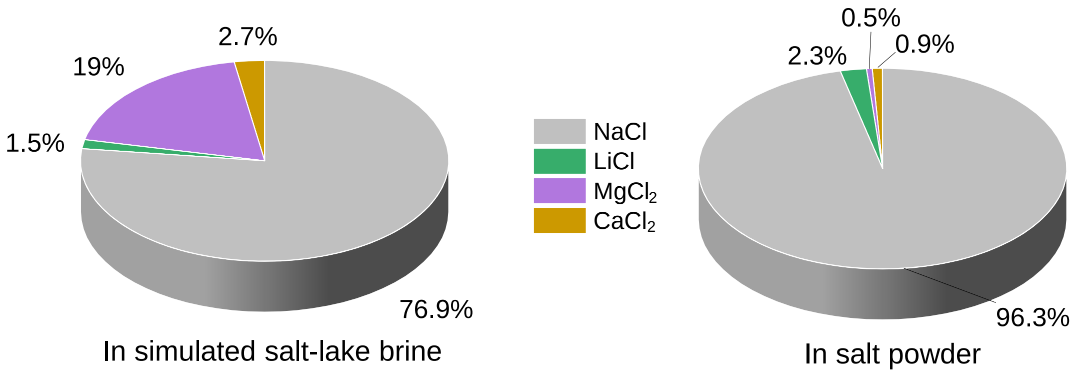

Supplementary Fig. 5 NaCl, LiCl, $MgCl_2$, and $CaCl_2$ proportion in simulated salt-lake brine and in the solid salt powder collected from the surface of the PANI nanoarrays solar evaporator.

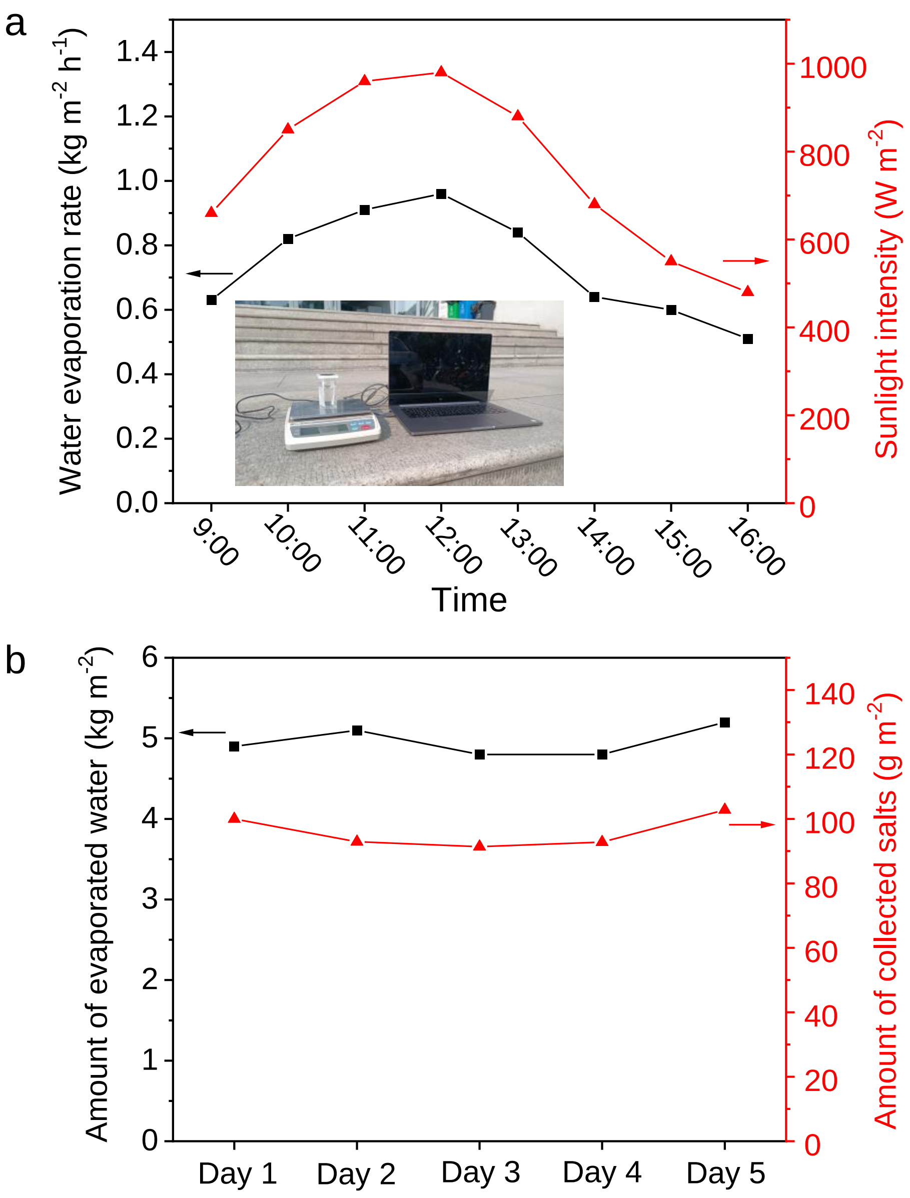

Supplementary Fig. 6 Outdoor experiments. (a) Sunlight intensity and water evaporation rate during the outdoor experiment from 9:00 to 16:00 at Soochow University campus during 25-29th October. Inset is the setup for the evaporation. (b) The amount of evaporated water and collected salt crystals on the unit area during the 5-day test.

Supplementary Table 1. Surface chemical components of the PA membranes.

Membrane	C (%)	Surface chemical species from C1*s*			N (%)	Surface chemical species from N1*s*			O (%)	Crosslinking degree (%)
		B.E. (eV)	Species	Content (%)		B.E. (eV)	Species	Content (%)		
PA-1	71.60	284.8	C-C/C-H	37.09	13.00	400.0	O=C-N	11.19	15.40	70.60
		286.0	C-N	25.31						
		287.7	O=C-N	6.35		401.7	N-H	**1.81**		
		288.5	O=C-O	**2.85**						
PA-2	71.77	284.8	C-C/C-H	37.25	12.60	400.0	O=C-N	11.15	15.63	74.09
		286.0	C-N	25.59						
		287.7	O=C-N	6.48		401.7	N-H	**1.45**		
		288.5	O=C-O	**2.45**						
PA-4	72.35	284.8	C-C/C-H	37.19	12.14	400.0	O=C-N	11.03	15.51	78.28
		286.0	C-N	25.95						
		287.7	O=C-N	7.26		401.7	N-H	**1.11**		
		288.5	O=C-O	**1.95**						

The crosslinking degree of PA-m was calculated by the following equation (2):

$$\text{Crosslinking degree} = \frac{amide\ links}{potential\ amide\ links} = \frac{N-AG}{N+CG} \quad (2)$$

Where *N*, *AG*, and *CG* represent the content of nitrogen, amine groups, and carboxylic groups respectively in the active layer.

Supplementary Table 2. The Salt concentration in simulated salt-lake brine and evaporated solution.

	NaCl	LiCl	$MgCl_2$	$CaCl_2$
Salt concentration in simulated salt-lake brine (g L^{-1})	268.0	5.1	66.1	9.2
Salt concentration in evaporated solution (g L^{-1})#	209.9	5.0	1.1	2.0
Rejection (%)	21.7	2.0	98.3	78.5

#Note: To determine the salt rejection, we assumed the salt powder was dissolved in the evaporated water.

Supplementary References

1. Zhu, Y.; Xie, W.; Gao, S.; Zhang, F.; Zhang, W.; Liu, Z.; Jin, J., Single-walled carbon nanotube film supported nanofiltration membrane with a nearly 10 nm thick polyamide selective layer for high-flux and high-rejection desalination. *Small* **2016,** *12* (36), 5034-5041.
2. Liang, Y.; Zhu, Y.; Liu, C.; Lee, K.-R.; Hung, W.-S.; Wang, Z.; Li, Y.; Elimelech, M.; Jin, J.; Lin, S., Polyamide nanofiltration membrane with highly uniform sub-nanometre pores for sub-1 Å precision separation. *Nat. Commun.* **2020,** *11* (1), 2015.
